# Ischemic Brain Lesions After Carotid Artery Stenting Increase Future Cerebrovascular Risk

**DOI:** 10.1016/j.jacc.2014.11.038

**Published:** 2015-02-17

**Authors:** Henrik Gensicke, H. Bart van der Worp, Paul J. Nederkoorn, Sumaira Macdonald, Peter A. Gaines, Aad van der Lugt, Willem P.Th.M. Mali, Philippe A. Lyrer, Nils Peters, Roland L. Featherstone, Gert J. de Borst, Stefan T. Engelter, Martin M. Brown, Leo H. Bonati, ICSS-MRI Substudy Investigators

**Affiliations:** ∗ Department of Neurology and Stroke Center, University Hospital Basel, Basel, Switzerland; † Department of Neurology and Neurosurgery, Brain Center Rudolf Magnus, University Medical Center Utrecht, Utrecht, the Netherlands; ‡ Department of Neurology, Academic Medical Center, Amsterdam, the Netherlands; § Department of Radiology, Freeman Hospital, Newcastle-upon-Tyne, United Kingdom; ‖ Sheffield Vascular Institute, Northern General Hospital, Sheffield, United Kingdom; ¶ Department of Radiology, Erasmus MC, University Medical Center Rotterdam, Rotterdam, the Netherlands; # Department of Radiology, University Medical Center Utrecht, Utrecht, the Netherlands; ∗∗ Department of Brain Repair and Rehabilitation, UCL Institute of Neurology, Queen Square, London, United Kingdom; †† Department of Vascular Surgery, University Medical Center Utrecht, Utrecht, the Netherlands

**Keywords:** carotid stenosis, DWI lesions, endarterectomy, long-term outcome, stenting, ARWMC, age-related white matter changes, CAS, carotid artery stenting, CEA, carotid endarterectomy, DWI, diffusion-weighted imaging, FLAIR, fluid-attenuated inversion recovery, MRI, magnetic resonance imaging, TIA, transient ischemic attack

## Abstract

**Background:**

Brain lesions on diffusion-weighted imaging (DWI) are frequently found after carotid artery stenting (CAS), but their clinical relevance remains unclear.

**Objectives:**

This study sought to investigate whether periprocedural ischemic DWI lesions after CAS or carotid endarterectomy (CEA) are associated with an increased risk of recurrent cerebrovascular events.

**Methods:**

In the magnetic resonance imaging (MRI) substudy of ICSS (International Carotid Stenting Study), 231 patients with symptomatic carotid stenosis were randomized to undergo CAS (n = 124) or CEA (n = 107). MRIs were performed 1 to 7 days before and 1 to 3 days after treatment. The primary outcome event was stroke or transient ischemic attack in any territory occurring between the post-treatment MRI and the end of follow-up. Time to occurrence of the primary outcome event was compared between patients with (DWI+) and without (DWI–) new DWI lesions on the post-treatment scan in the CAS and CEA groups separately.

**Results:**

Median time of follow-up was 4.1 years (interquartile range: 3.0 to 5.2). In the CAS group, recurrent stroke or transient ischemic attack occurred more often among DWI+ patients (12 of 62) than among DWI– patients (6 of 62), with a cumulative 5-year incidence of 22.8% (standard error [SE]: 7.1%) and 8.8% (SE: 3.8%), respectively (unadjusted hazard ratio: 2.85; 95% confidence interval: 1.05 to 7.72; p = 0.04). In DWI+ and DWI– patients, 8 and 2 events, respectively, occurred within 6 months after treatment. In the CEA group, there was no difference in recurrent cerebrovascular events between DWI+ and DWI– patients.

**Conclusions:**

Ischemic brain lesions discovered on DWI after CAS seem to be a marker of increased risk for recurrent cerebrovascular events. Patients with periprocedural DWI lesions might benefit from more aggressive and prolonged antiplatelet therapy after CAS. (A Randomised Comparison of the Risks, Benefits and Cost Effectiveness of Primary Carotid Stenting With Carotid Endarterectomy: International Carotid Stenting Study; ISRCTN25337470)

The occurrence of periprocedural ischemic brain lesions on magnetic resonance imaging (MRI) after revascularization of atherosclerotic stenosis of the internal carotid artery, either with stenting (CAS) or endarterectomy (CEA), has been commonly described (1). The randomized ICSS (International Carotid Stenting Study) compared CAS with CEA in patients with symptomatic carotid stenosis (2). In the MRI substudy of ICSS (ICSS-MRI), 50% of patients treated with CAS and 17% of those undergoing CEA had periprocedural ischemic brain lesions on diffusion-weighted imaging (DWI) on MRI scans obtained a median of 1 day after treatment (adjusted odds ratio 5.21; 95% confidence interval [CI]: 2.78 to 9.79; p < 0.0001) (3). However, the clinical significance of these lesions remains unclear. Previous research focused mainly on the persistence of lesions on follow-up imaging 4, 5, 6, 7 and their effects on neuropsychological function 8, 9, 10.

The goal of the present analysis of the ICSS-MRI substudy was to investigate whether the occurrence of periprocedural DWI lesions altered the risk of future cerebrovascular events during long-term follow-up.

## Methods

The prospective multicenter ICSS-MRI substudy included 124 patients randomly assigned to CAS and 107 patients randomly assigned to CEA in ICSS. The study design and the main short- and long-term results of ICSS and the ICSS-MRI substudy have been reported previously 2, 3, 11. Briefly, ICSS patients with recently symptomatic moderate or severe carotid stenosis (defined by a luminal narrowing of ≥50% according to the measurement of degree of stenosis used in the North American Symptomatic Carotid Endarterectomy Trial [12]) were randomized in a 1:1 ratio to receive CAS or CEA. Baseline imaging of the target artery was specified to require consistent findings on at least 2 noninvasive imaging modalities, including computed tomography angiography, magnetic resonance angiography, and duplex ultrasound; or intra-arterial digital subtraction angiography. Eight patients in the CAS group and 7 patients in the CEA group underwent digital subtraction angiography before the randomly allocated procedure, and the remainder received noninvasive imaging. The protocol recommended the use of a cerebral protection device during CAS whenever such a device could be safely deployed, but this action was not mandatory. The combination of aspirin and clopidogrel was recommended to cover the period of stenting and to be continued for a minimum of 4 weeks after the procedure.

**Figure 1:**
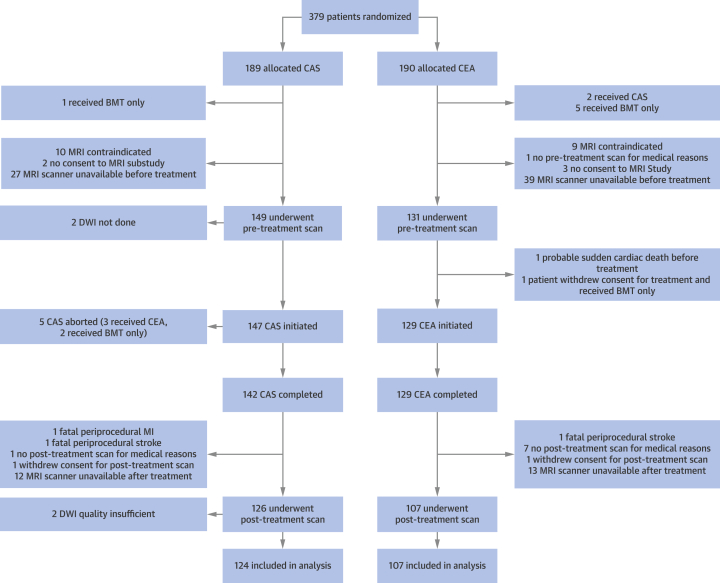
Study Flow Diagram Diagram outlining the 2 arms of the study, including events that precluded patients from analysis. Scans are magnetic resonance imaging (MRI). BMT = best medical treatment; CAS = carotid artery stenting; CEA = carotid endarterectomy; DWI = diffusion-weighted imaging; MI = myocardial infarction.

If no contraindications to MRI were present, all patients included at 7 ICSS centers had the option of participating in the ICSS-MRI substudy (Figure 1). MRI scans at field strengths of 1.5- or 3-T (including DWI and fluid-attenuated inversion recovery [FLAIR] sequences) were specified to be conducted 1 to 7 days before treatment (pre-treatment MRI) and 1 to 3 days after treatment (post-treatment MRI). Assessment of MRI scans was performed through consensus reading by a neurologist (L.H.B.) and a neuroradiologist (L.M.J.) who were blinded to treatment allocation and clinical outcome. In cases of disagreement between the 2 reviewers, a third reviewer (S.T.E.) made the final decision. New periprocedural ischemic brain lesions were defined as hyperintense DWI lesions on the post-treatment MRI that were not present on the pre-treatment MRI. Furthermore, the number (lesion count) and total and individual volumes of new DWI lesions were assessed in each patient. Age-related white matter changes (ARWMC) on the baseline scan were quantified on pre-treatment FLAIR sequences by using a published scale (13).

**Figure 2:**
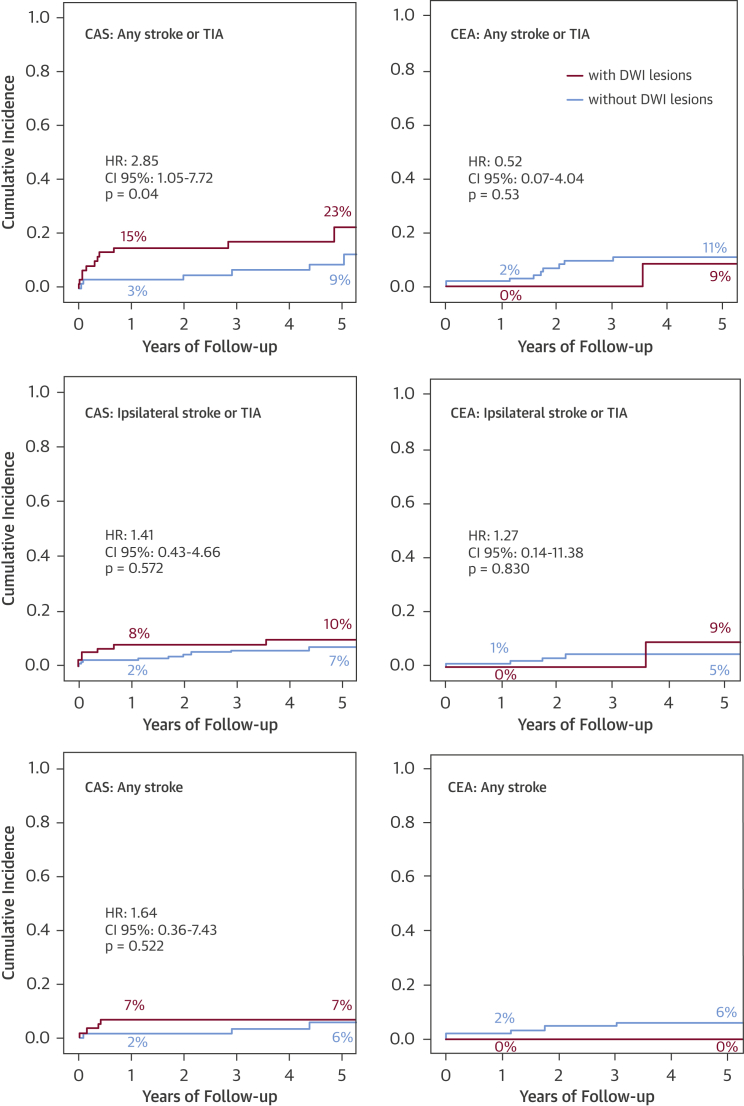
Cumulative Incidence Rates of Outcome Events During Follow-Up Comparison of any stroke or transient ischemic attack (TIA), ipsilateral stroke or TIA, and any stroke between patients with periprocedural DWI lesions **(red curve)** and without periprocedural DWI lesions **(blue curve)** with either carotid artery stenting (CAS) or carotid endarterectomy (CEA). Percentages are point estimates of cumulative incidences after 1 and 5 years of follow-up. CI = confidence interval; HR = hazard ratio.

In ICSS, patients were followed up for 30 days after treatment and then at 6 months and annually after randomization by clinicians who were not involved in delivering the treatment. Outcome events were recorded at the centers, reported to the study office, and centrally adjudicated by the chief investigator. An independent external adjudicator who was blinded to treatment allocation also received the outcome events, including death or stroke.

The primary combined outcome event of the present analysis was stroke or transient ischemic attack (TIA) located in any vascular territory, occurring between the post-treatment MRI scan and the end of available follow-up. Stroke was defined as a rapidly developing clinical syndrome of focal disturbance of cerebral function lasting >24 h or leading to death with no apparent cause other than that of vascular origin. TIA was defined as a rapidly developing clinical syndrome of focal disturbance of cerebral function lasting <24 h with no apparent cause other than cerebral ischemia. Secondary outcome events were stroke or TIA occurring in the territory supplied by the treated carotid artery and stroke alone occurring in any territory.

Patients in the CAS group and the CEA group were analyzed separately. The relation between new periprocedural DWI lesions and time until occurrence of cerebrovascular events during follow-up was analyzed in 2 different ways: first, by the presence (DWI+) or absence (DWI–) of periprocedural DWI lesions (binary analysis); and second, by the absolute number of periprocedural DWI lesions (count analysis). The following baseline variables were assessed for adjustment of the analyses: age, sex, history of vascular risk factors (any smoking [current or past], diabetes, hypertension, hyperlipidemia, coronary heart disease, or peripheral artery disease), systolic blood pressure, total serum cholesterol, modified Rankin Scale (14), type of qualifying event (defined as the most recent ipsilateral ischemic event before randomization [retinal ischemia, TIA, or hemispheric stroke]), the ARWMC sum score (13), degree of ipsilateral and contralateral stenosis (mild 30% to 49%; moderate 50% to 69%; and severe 70% to 99%), and the presence or absence of hyperintense DWI lesions on pre-treatment MRI.

### Standard protocol approvals, registrations, and patient consent

The North West Multi-Centre Research Ethics Committee in the United Kingdom and local ethics committees for non–United Kingdom centers approved the study. All patients provided written informed consent.

### Statistical analysis

Demographic and clinical baseline characteristics were compared between patients with and without new DWI lesions after treatment by using the Fisher exact test or the chi-square test as appropriate for categorical variables and the Mann-Whitney *U* test for continuous variables. In the binary analysis, we compared the time until occurrence of an outcome event between DWI+ and DWI– patients (with the latter as the reference group) by using Cox proportional hazards regression models to calculate hazard ratios (HRs) with 95% CIs. The proportional hazards assumption was checked with test results and diagnostic plots on the basis of scaled Schoenfeld residuals. In the lesion count analysis, the association between the number of new periprocedural DWI lesions and time until occurrence of an outcome event were analyzed by using Cox regression models. In the case of occurrence of multiple strokes or TIAs, only the first event qualifying as an outcome event was counted. Kaplan-Meier statistics were used to estimate the cumulative incidences of outcome events and their standard errors (SEs) at 1 and 5 years after the post-treatment MRI scan. Models were adjusted for the clinical or demographic variable that was most significantly associated with time until occurrence of a clinical outcome event in univariate testing, provided that the p value of the univariate association was <0.1.

## Results

The present analysis of the ICSS-MRI substudy included the same population as previously reported (Figure 1) (3). A total of 231 patients were randomly allocated to receive CAS (n = 124) or CEA (n = 107). Sixty-six patients were studied in 3-T scanners (CAS, n = 37; CEA, n = 29) and 165 patients in 1.5-T scanners. In the CAS group, 51 patients were treated with cerebral protection devices, whereas 73 patients underwent unprotected stenting.

In the CAS group, 62 (50%) of 124 patients had new ischemic DWI lesions on post-treatment MRI. Patients with DWI+ were older by a median of 5 years, smoked less often, had lower total cholesterol levels, and had a higher median ARWMC score at randomization (Table 1). During a median follow-up of 4.65 years (interquartile range: 2.97 to 5.28 years), 10 patients died in the DWI+ group (cumulative 5-year mortality rate 16.5% [SE: 6.3%]), and 10 patients died in the DWI– group (15.5% [SE: 4.8%]). Table 2 summarizes medical treatment before the procedure and during follow-up.

**Table 1:** Characteristics of Patients With and Without Periprocedural DWI Lesions in the Stenting and Endarterectomy Groups

	Stenting			Endarterectomy		
	DWI+ (n = 62 [50%])	DWI– (n = 62 [50%])	p Value	DWI+ (n = 18 [16.8%])	DWI– (n = 89 [83.2%])	p Value
Age, yrs	74 (68–80)	69 (60–75)	**0.003**	70 (64–74)	72 (63–76)	0.727
Male	42 (67.7)	45 (72.6)	0.566	13 (72.7)	63 (70.8)	0.903
Vascular risk factors						
Smoking	42 (67.7)	52 (83.9)	**0.036**	13 (72.2)	67 (75.3)	0.785
Diabetes	14 (22.6)	10 (16.1)	0.367	3 (16.7)	21 (23.6)	0.520
Hypertension	47 (75.8)	38 (61.3)	0.083	12 (66.7)	62 (69.7)	0.802
Hyperlipidemia	42 (67.7)	36 (58.1)	0.268	12 (66.7)	60 (67.4)	0.951
CHD	18 (29.0)	12 (19.4)	0.212	4 (22.2)	17 (19.1)	0.761
PAD	8 (12.9)	14 (22.6)	0.161	2 (11.1)	13 (14.6)	0.697
Systolic blood pressure at randomization, mm Hg	160 (135–175)	160 (133–180)	0.985	165 (150–180)	155 (140–170)	0.069
Total cholesterol at randomization, mmol/l	4.3 (3.4–5.0)	5.0 (4.2–6.0)	**<0.001**	5.1 (4.3–6.1)	4.8 (4.1–5.6)	0.515
Baseline mRS			0.301			0.229
0	26 (41.9)	28 (45.2)		10 (55.6)	28 (31.5)	
1	17 (27.4)	12 (19.4)		5 (27.8)	24 (27.0)	
2	16 (25.8)	15 (24.2)		3 (16.7)	25 (28.1)	
3	1 (1.6)	6 (9.7)		0 (0.0)	9 (10.1)	
4	2 (3.2)	1 (1.6)		0 (0.0)	3 (3.4)	
Qualifying event			0.136			0.363
Retinal ischemia	11 (17.7)	16 (25.8)		4 (22.2)	18 (20.2)	
TIA	18 (29.0)	24 (38.7)		10 (55.6)	36 (40.4)	
Stroke	33 (53.2)	22 (35.5)		4 (22.2)	35 (39.3)	
ARWMC score	5 (3–9)	4 (1–7)	**0.011**	4 (3–8)	4 (2–8)	0.763
Stenosis, ipsilateral			0.433			0.734
Moderate (50%–69%)	10 (16.1)	7 (11.8)		1 (5.6)	7 (7.9)	
Severe (70%–99%)	52 (83.9)	55 (88.7)		17 (94.4)	82 (92.1)	
Stenosis, contralateral			0.799			0.860
Mild (<50%)	42 (67.7)	37 (59.7)		13 (72.2)	61 (68.5)	
Moderate (50%–69%)	5 (8.1)	7 (11.3)		2 (11.2)	14 (15.7)	
Severe (70%–99%)	11 (17.7)	14 (22.6)		3 (16.7)	12 (13.5)	
Occlusion	4 (6.5)	4 (6.5)		0 (0.0)	2 (2.2)	

Values are median (interquartile range) or n (%). Values in **bold** are <0.05.

ARWMC = age-related white matter changes; CHD = coronary heart disease; DWI+ = presence of periprocedural diffusion-weighted imaging lesions; DWI– = absence of periprocedural diffusion-weighted imaging lesions; mRS = modified Rankin Scale; PAD = peripheral artery disease; TIA = transient ischemic attack.

**Table 2:** Medical Treatment Before Procedure and During Follow-Up

	Before Procedure		After 6 Months		After 1 Year		After 5 Years	
	CAS (n = 118)	CEA (n = 102)	CAS (n = 112)	CEA (n = 102)	CAS (n = 104)	CEA (n = 92)	CAS (n = 55)	CEA (n = 42)
Any antiplatelet therapy	118 (100)	95 (93)	109 (97)	93 (91)	99 (95)	85 (92)	51 (93)	38 (90)
Aspirin	111 (94)	87 (85)	102 (91)	86 (84)	87 (84)	70 (76)	48 (87)	33 (78.6)
Clopidogrel	114 (97)	13 (12)	5 (5)	11 (11)	7 (7)	9 (10)	1 (2)	4 (10)
Dipyridamole + aspirin	15 (13)	27 (27)	20 (18)	15 (15)	23 (22)	16 (17)	13 (24)	10 (24)
Dual antiplatelet therapy	109 (92)	6 (6)	8 (7)	5 (5)	5 (5)	5 (5)	1 (2)	1 (2)
Anticoagulation (vitamin K antagonists)	4 (3)	6 (6)	3 (3)	6 (6)	2 (2)	7 (8)	1 (2)	4 (10)
Any anticoagulation or antiplatelet	118 (100)	100 (98)	110 (98)	99 (97)	101 (97)	90 (98)	51 (93)	41 (98)
Antihypertensive therapy	–	–	79 (71)	74 (73)	71 (68)	69 (75)	44 (80)	30 (71)
Lipid lowering therapy	–	–	94 (84)	83 (81)	85 (82)	76 (83)	41 (75)	32 (76)

Values are n with available data (%).

CAS = carotid artery stenting; CEA = carotid endarterectomy.

In the CAS group, recurrent stroke or TIA in any territory occurred more often among DWI+ patients (12 of 62) than among DWI– patients (6 of 62) in the binary analysis (unadjusted HR: 2.85; 95% CI: 1.05 to 7.72; p = 0.04) (Table 3, Central Illustration). Among the tested baseline variables, systolic blood pressure at randomization was associated with stroke or TIA in any territory on the univariate level at a p value <0.1 (Online Table 1). The association between periprocedural DWI lesions and recurrent stroke or TIA remained significant after adjustment for systolic blood pressure (adjusted HR: 3.52; 95% CI: 1.21 to 10.22; p = 0.021). The cumulative probability of having a stroke or TIA in the CAS group was 15.1% (SE: 4.7%) in DWI+ patients compared with 3.2% (SE: 2.2%) in DWI– patients at 1 year, and 22.8% (SE: 7.1%) compared with 8.8% (SE: 3.8%), respectively, at 5 years. The cumulative incidence curves particularly diverged during the first 6 months after the post-treatment scan, when 8 of 12 events occurred in DWI+ patients and 2 of 6 events occurred in DWI– patients.

**Table 3:** HRs of Outcome Events During Follow-Up Depending on Post-Treatment DWI Lesions

	Stroke or TIA in Any Territory	Ipsilateral Stroke or TIA	Stroke in Any Territory
Stenting (n = 124)			
DWI+ (n = 62) vs. DWI− (n = 62)∗			
Unadjusted	2.85 (1.05–7.72), **p = 0.040**	1.41 (0.43–4.66), p = 0.572	1.64 (0.36–7.43), p = 0.522
Adjusted	3.52 (1.21–10.22), **p = 0.021**	3.15 (0.90–11.10), p = 0.073	
DWI lesion count†			
Unadjusted	1.03 (1.01–1.04), **p = 0.003**	1.02 (1.00–1.05), **p = 0.020**	1.03 (1.01–1.06), **p = 0.003**
Adjusted	1.03 (1.01–1.05), **p = 0.001**	1.03 (1.01–1.05), **p = 0.014**	
Bootstrap CI‡	1.00–1.05	0.98–1.05	0.99–1.07
Endarterectomy (n = 107)			
DWI+ (n = 18) vs. DWI− (n = 89)∗			
Unadjusted	0.52 (0.07–4.04), p = 0.528	1.27 (0.14–11.38), p = 0.830	—§
Adjusted	0.59 (0.74–4.67), p = 0.616	1.55 (0.16–14.96), p = 0.706	
DWI lesion count†			
Unadjusted	1.08 (0.89–1.32), p = 0.433	1.18 (0.97–1.43), p = 0.097	0.48 (0.03–8.83), p = 0.619
Adjusted	1.08 (0.98–1.28), p = 0.572	1.14 (0.92–1.41), p = 0.228	

Values are HR (95% CI). Values in **bold** are <0.05.

Abbreviations as in Table 1.

∗ Cox hazard ratios (HRs), 95% confidence intervals (CIs), and p values for outcome events during follow-up in patients with periprocedural DWI lesions versus those without.

† Cox HRs, 95% CIs, and p values for outcome events during follow-up depending on the number of periprocedural DWI lesions (DWI lesion count).

‡ Bootstrap CIs were calculated with R statistical software by using a bias-corrected method.

§ HR not calculated as no strokes occurred in group with periprocedural DWI lesions.

**Central Illustration:**
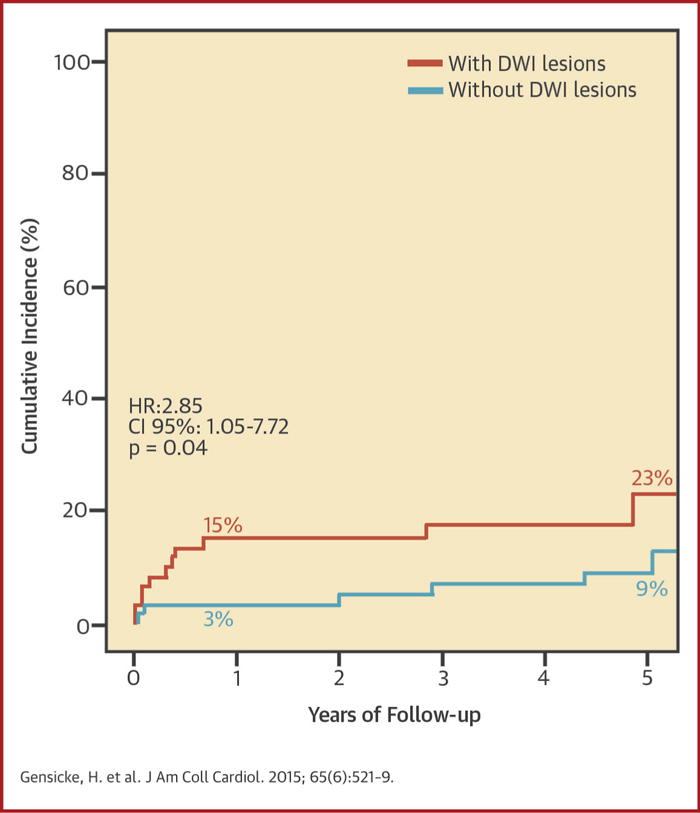
Significance of Brain Lesions After Carotid Artery Stenting: Kaplan-Meier Cumulative Incidence Curves of Recurrent Stroke or TIA During Follow-Up Unadjusted Cox regression hazard ratio (HR) for any stroke or TIA in patients with and without new ischemic diffusion-weighted imaging (DWI) lesions on post-treatment magnetic resonance imaging. Percentages are point estimates of cumulative incidences after 1 and 5 years of follow-up. CI = confidence interval.

Between DWI+ and DWI– patients in the CAS group, there was no significant difference in the risk of ipsilateral stroke or TIA or in the risk of stroke alone (Table 3, Figure 2). However, in the lesion count analysis, a higher number of periprocedural DWI lesions were significantly associated with all 3 outcome events in the CAS group, both before and after adjustment for baseline variables (Table 3, Online Table 1). To investigate whether the Cox regression models were overly influenced by a small number of patients with high DWI lesion counts, we performed bootstrapping of HR CIs with 100,000 iterations by using the bias-corrected method with R statistical software (R Foundation for Statistical Computing, Vienna, Austria). Although the results displayed some evidence of overinfluencing, the model with the primary outcome measure (i.e., any stroke or TIA) remained significant.

In the CEA group, 18 (16.8%) patients had new ischemic DWI lesions on post-treatment MRI, whereas 89 (83.2%) patients had no lesions. Baseline characteristics did not differ significantly between patients with and without lesions (Table 1). During a median follow-up of 4.02 years (interquartile range: 2.93 to 5.11), 1 patient in the DWI+ group died (cumulative 5-year mortality rate 5.9% [SE: 5.7%]) and 10 patients in the DWI– group died (19.0% [SE: 5.8%]). There was no significant association between the presence or number of periprocedural DWI lesions and any outcome measures in the binary analysis or in the count analysis (Table 3, Figure 2).

None of the recurrent events observed in the ICSS-MRI substudy were associated with residual or recurrent carotid stenosis after treatment, and none of the patients with recurrent events underwent repeat revascularization by CAS or CEA.

## Discussion

The present study revealed that in patients treated with CAS: 1) the occurrence of periprocedural ischemic brain lesions on DWI increased the risk of recurrent stroke or TIA in any territory during follow-up; 2) most of these events occurred within the first 6 months after treatment; 3) a higher count of periprocedural DWI lesions additionally increased the risk of ipsilateral stroke or TIA and any stroke; and 4) in patients undergoing CEA, neither the presence nor the number of periprocedural DWI lesions was associated with the risk of future cerebrovascular events.

Previously, several studies found a higher frequency of new ischemic brain lesions on DWI after CAS than after CEA (1). In a meta-analysis of nonrandomized and randomized studies, the odds of detecting new lesions after treatment were increased after CAS compared with CEA by a ratio of 6.16 (95% CI: 4.45 to 8.54) (3). The proportion of patients with periprocedural DWI lesions after CAS was 50% in the ICSS-MRI substudy but has been reported in the literature to be as high as 87.1% (15). Despite the frequent occurrence of these lesions, their significance for individual patients remains unclear. Previous analyses of the ICSS-MRI substudy revealed that CAS patients had more lesions persisting on FLAIR imaging 1 month after treatment than CEA patients but that the rate of conversion from acute to persisting lesions was lower in the CAS group than in the CEA group; this finding was attributed to the smaller size of the individual lesions in the CAS group (7). A single-center substudy in ICSS detected a small but statistically significant decrease in global cognitive performance 6 months after CAS compared with baseline values, which was not present in the CEA group. However, cognitive decline was not associated with the occurrence of periprocedural DWI lesions, although the study was likely underpowered to rule out such an association (8).

We are unaware of any previous studies investigating the impact of periprocedural DWI lesions in carotid revascularization on the risk of future cerebrovascular events. Our findings lend support to the hypothesis that periprocedural DWI lesions seen after CAS may be a marker of unstable atherosclerotic plaques, which increase the risk of cerebral embolism not only during the procedure but also in the first few months thereafter. DWI lesions and ischemic complications during stent placement are more common among patients with unstable plaques characterized by echolucent appearance on ultrasound and lipid-rich necrotic plaque and intraplaque hemorrhage on MRI 16, 17, 18. Another study showed that once atherothrombosis has occurred, the plaque may remain chronically unstable unless trigger factors such as inflammation or increased shear stress are removed (19). Unstable plaques might not only be present in the target artery but also in the access vasculature, including the aortic arch. Aortic arch lesions have been shown to increase the risk of cerebral embolism during CAS 20, 21. Unstable aortic atheroma causing delayed embolism may explain why recurrent events also occurred outside the territory supplied by the treated carotid artery in the present study.

The curves of event rates seen in our study may indicate that patients with periprocedural DWI lesions after CAS remain at increased risk of cerebrovascular events for the first 6 months after treatment. This finding might support more aggressive and prolonged antiplatelet treatment among such patients. More aggressive antiplatelet regimens may also prevent cerebral ischemia occurring as a result of the procedure. In a recent randomized study (22), an increase in the clopidogrel loading dose from 300 to 600 mg administered before the intervention significantly reduced the incidence of the combined outcome event of new DWI lesions on post-treatment MRI or any stroke or TIA occurring within 30 days of treatment. In the ICSS-MRI substudy, 92% of patients in the CAS group received dual antiplatelet therapy before the procedure, but loading doses were not recorded. In a post-hoc analysis, we found no significant difference in the risk of periprocedural DWI lesions between patients undergoing CAS under monotherapy versus dual antiplatelet therapy, but our study was not powered to demonstrate such a difference. As shown by our follow-up data regarding medication, dual antiplatelet therapy was not usually given for a prolonged period of time beyond the recommended 4 weeks after the procedure.

Because routine DWI scanning after CAS increases costs, and given the high incidence of new periprocedural brain lesions, there may be justification for administering more aggressive and prolonged antiplatelet therapy to all patients undergoing CAS. However, our results need to be confirmed in larger studies before such recommendations can be made.

### Study limitations

First, both 1.5- and 3-T scanners were used in the study. Differences in magnetic field strengths may have led to differences in the sensitivity of detecting small ischemic brain lesions on DWI. Second, the absolute number of strokes occurring in our study was small, which is why we chose stroke or TIA as the combined primary outcome event. For the patient, TIA is less relevant an outcome than stroke. However, our analysis of DWI lesion count indicated that the presence of a large number of these lesions, which as previously reported were often small and clinically silent in the majority of patients 3, 23, seems to be associated with a higher risk for recurrent stroke as well. Third, the small number of outcome events did not allow differentiation between the effects of periprocedural DWI lesions on recurrent events occurring inside versus outside the vascular territory supplied by the treated carotid artery. Finally, the small number of patients with new periprocedural DWI lesions after CEA precluded a meaningful analysis of the impact of these lesions on future risk of cerebrovascular events in the surgical arm.

## Conclusions

Ischemic brain lesions discovered on DWI after CAS seem to be a marker of increased risk for recurrent cerebrovascular events. Patients with periprocedural DWI lesions might benefit from more aggressive and prolonged antiplatelet therapy after CAS.

Perspectives **COMPETENCY IN MEDICAL KNOWLEDGE:** Ischemic brain lesions are often found on MRI after stenting of atherosclerotic carotid stenosis; most are clinically asymptomatic, but they identify patients at increased risk of future stroke or TIA.

**TRANSLATIONAL OUTLOOK:** Because most cerebral ischemic events present clinically within the first 6 months after carotid stenting, future trials should address whether patients with asymptomatic post-procedural ischemia benefit from more prolonged dual antiplatelet therapy.

## Appendix

ICSS-MRI Substudy Investigators and Online Table 1

## References

[1] Schnaudigel S., Groschel K., Pilgram S.M., Kastrup A. (2008). New brain lesions after carotid stenting versus carotid endarterectomy: a systematic review of the literature. Stroke.

[2] Bonati L.H., Dobson J., Featherstone R.L. (2014 Oct 14). Long-term outcomes after stenting versus endarterectomy for treatment of symptomatic carotid stenosis: the International Carotid Stenting Study (ICSS) randomised trial. Lancet.

[3] Bonati L.H., Jongen L.M., Haller S. (2010). New ischaemic brain lesions on MRI after stenting or endarterectomy for symptomatic carotid stenosis: a substudy of the International Carotid Stenting Study (ICSS). Lancet Neurol.

[4] Hauth E.A., Jansen C., Drescher R. (2005). MR and clinical follow-up of diffusion-weighted cerebral lesions after carotid artery stenting. Am J Neuroradiol.

[5] Palombo G., Faraglia V., Stella N., Giugni E., Bozzao A., Taurino M. (2008). Late evaluation of silent cerebral ischemia detected by diffusion-weighted MR imaging after filter-protected carotid artery stenting. Am J Neuroradiol.

[6] Zhou W., Dinishak D., Lane B., Hernandez-Boussard T., Bech F., Rosen A. (2009). Long-term radiographic outcomes of microemboli following carotid interventions. J Vasc Surg.

[7] Rostamzadeh A., Zumbrunn T., Jongen L.M. (2014). Predictors of acute and persisting ischemic brain lesions in patients randomized to carotid stenting or endarterectomy. Stroke.

[8] Altinbas A., van Zandvoort M.J., van den B.E. (2011). Cognition after carotid endarterectomy or stenting: a randomized comparison. Neurology.

[9] Lal B.K., Younes M., Cruz G., Kapadia I., Jamil Z., Pappas P.J. (2011). Cognitive changes after surgery vs stenting for carotid artery stenosis. J Vasc Surg.

[10] Wasser K., Pilgram-Pastor S.M., Schnaudigel S. (2011). New brain lesions after carotid revascularization are not associated with cognitive performance. J Vasc Surg.

[11] Ederle J., Dobson J., Featherstone R.L. (2010). Carotid artery stenting compared with endarterectomy in patients with symptomatic carotid stenosis (International Carotid Stenting Study): an interim analysis of a randomised controlled trial. Lancet.

[12] (1991). North American Symptomatic Carotid Endarterectomy Trial. Methods, patient characteristics, and progress. Stroke.

[13] Wahlund L.O., Barkhof F., Fazekas F. (2001). A new rating scale for age-related white matter changes applicable to MRI and CT. Stroke.

[14] van Swieten J.C., Koudstaal P.J., Visser M.C., Schouten H.J., van Gijn J. (1988). Interobserver agreement for the assessment of handicap in stroke patients. Stroke.

[15] Bijuklic K., Wandler A., Hazizi F., Schofer J. (2012). The PROFI study (Prevention of Cerebral Embolization by Proximal Balloon Occlusion Compared to Filter Protection During Carotid Artery Stenting): a prospective randomized trial. J Am Coll Cardiol.

[16] Yamada K., Yoshimura S., Kawasaki M. (2011). Embolic complications after carotid artery stenting or carotid endarterectomy are associated with tissue characteristics of carotid plaques evaluated by magnetic resonance imaging. Atherosclerosis.

[17] Tanemura H., Maeda M., Ichikawa N. (2013). High-risk plaque for carotid artery stenting evaluated with 3-dimensional T1-weighted gradient echo sequence. Stroke.

[18] Burow A., Lyrer P.A., Nederkoorn P.J. (2014). Echographic risk index and cerebral ischemic brain lesions in patients randomized to stenting versus endarterectomy for symptomatic carotid artery stenosis. Ultraschall Med.

[19] Spagnoli L.G., Mauriello A., Sangiorgi G. (2004). Extracranial thrombotically active carotid plaque as a risk factor for ischemic stroke. JAMA.

[20] Faggioli G., Ferri M., Rapezzi C., Tonon C., Manzoli L., Stella A. (2009). Atherosclerotic aortic lesions increase the risk of cerebral embolism during carotid stenting in patients with complex aortic arch anatomy. J Vasc Surg.

[21] Kastrup A., Groschel K., Schnaudigel S., Nagele T., Schmidt F., Ernemann U. (2008). Target lesion ulceration and arch calcification are associated with increased incidence of carotid stenting-associated ischemic lesions in octogenarians. J Vasc Surg.

[22] Patti G., Tomai F., Melfi R. (2013). Strategies of clopidogrel load and atorvastatin reload to prevent ischemic cerebral events in patients undergoing protected carotid stenting. Results of the randomized ARMYDA-9 CAROTID (Clopidogrel and Atorvastatin Treatment During Carotid Artery Stenting) study. J Am Coll Cardiol.

[23] Gensicke H., Zumbrunn T., Jongen L.M. (2013). Characteristics of ischemic brain lesions after stenting or endarterectomy for symptomatic carotid artery stenosis: results from the International Carotid Stenting Study-Magnetic Resonance Imaging substudy. Stroke.

