# Supplementary material for: Ischemic Brain Lesions After Carotid Artery Stenting Increase Future Cerebrovascular Risk
Source: J Am Coll Cardiol. 2015 Feb 17;65(6):521–9. doi: 10.1016/j.jacc.2014.11.038 (PMC4323145; doi:10.1016/j.jacc.2014.11.038)
Supplement: ICSS-MRI Substudy Investigators and Online Table 1 [file mmc1.docx]

**Online Appendix**

**ICSS-MRI Study centres and collaborators**

The following centres enrolled patients in the ICSS-MRI Study [number of patients included in primary analysis per centre].

**University Medical Centre**, Utrecht, The Netherlands [129]: GJ de Borst, GAP de Kort, LM Jongen, LJ Kappelle, TH Lo, WPThM Mali, FL Moll, HB van der Worp.

**University Hospital Basel**, Switzerland [50]: LH Bonati, ST Engelter, F Fluri, S Haller, AL Jacob, E Kirsch, PA Lyrer, E-W Radue, P Stierli, M Wasner, SG Wetzel.

**Erasmus Medical Centre**, Rotterdam, The Netherlands [33]: HZ Flach, JM Hendriks, PJ Koudstaal, PMT Pattynama, LC van Dijk, MRHM van Sambeek, A van der Lugt, H van Urk, HJM Verhagen.

**Newcastle Acute Hospitals NHS Foundation Trust**, Newcastle-Upon-Tyne, UK [8]: M Clarke, M Davis, AK Dixit, P Dorman , A Dyker, G Ford, A Golkar, R Jackson, V Jayakrishnan, D Lambert, T Lees, S Louw, S Macdonald, D Mendelow, H Rodgers, J Rose, G Stansby, M Wyatt.

**Academic Medical Centre**, Amsterdam, The Netherlands [5]: MJW Koelemaij, CBLM Majoie, PJ Nederkoorn, JAA Reekers, YBWEM Roos.

**University College London Hospitals NHS Foundation Trust**, London, UK [5]: M Adiseshiah, C Bishop, S Brew, J Brookes, MM Brown, HR Jäger, N Kitchen.

**Sheffield Teaching Hospitals NHS Foundation Trust**, Sheffield, UK [1]: J Beard, T Cleveland, D Dodd, PA Gaines, R Lonsdale, R Nair, A Nassef, S Nawaz, G Venables.

**Online Table 1. Univariate associations between clinical characteristics and cerebrovascular events during follow-up**

|  | Stenting | | | Endarterectomy | | |
| --- | --- | --- | --- | --- | --- | --- |
|  | Stroke or TIA in Any Territory | Ipsilateral Stroke  or TIA | Stroke  in Any Territory | Stroke or TIA in Any Territory | Ipsilateral Stroke  or TIA | Stroke  in Any Territory |
| Age | 0.99 (0.94-1.04), 0.673 | 0.98 (0.92-1.41), 0.475 | 1.02 (0.94-1.10), 0.650 | **1.09 (1.004-1.18), 0.038** | 1.08 (0.96-1.21), 0.223 | 1.05 (0.94-1.17), 0.412 |
| Female sex | 1.09 (0.41-2.90), 0.87 | 0.87 (0.23-3.27), 0.833 | 0.92 (0.18-4.73), 0.917 | 2.35 (0.72-7.71), 0.160 | 3.93 (0.66-23.53), 0.134 | 1.74 (0.29-10.43), 0.543 |
| Smoking | 1.10 (0.36-3.33), 0.872 | 3.14 (0.40-24.53), 0.275 | 0.74 (0.14-3.83), 0.722 | 0.87 (0.23-3.30), 0.843 | 0.49 (0.08-2.96), 0.439 | 1.40 (0.16-12.49), 0.766 |
| Diabetes | 0.58 (0.13-2.50), 0.461 | 0.99 (0.21-4.60), 0.992 | 0.73 (0.09-6.05), 0.768 | 1.74 (0.45-6.46), 0.433 | 1.03 (0.12-9.23), 0.979 | 2.64 (0.44-15.84), 0.288 |
| Hypertension | 0.83 (0.32-2.16), 0.700 | 1.28 (0.34-4.82), 0.718 | 39.96 (0.06-27261), 0.268 | 1.002 (0.28-3.54), 0.997 | 1.92 (0.21-17.19), 0.560 | 1.93 (0.22-17.31), 0.556 |
| Hyperlipdemia | 1-01 (0.39-2.61), 0.985 | 1.66 (0.44-6.25), 0.458 | 0.83 (0.19-3.72), 0.809 | 2.22 (0.47-10.27), 0.309 | 0.68 (0.11-4.09), 0.676 | 37.91 (0.015-9.4x10^4^), 0.362 |
| CHD | 1.25 (0.44-3.50), 0.676 | 1.21 (0.32-4.54), 0.783 | 1.28 (0.25-6.57), 0.771 | 0.96 (0.21-4.47), 0.960 | 2.79 (0.47-16.74), 0.261 | 0.04 (0.00-469.5), 0.490 |
| PAD | 1.33 (0.44-4.04), 0.620 | **2.84 (0.83-9.72), 0.096** | 1.86 (0.36-9.60), 0.461 | 0.65 (0.08-5.09), 0.677 | 1.48 (0.17-13.27), 0.725 | 0.04 (0.00-2085), 0.560 |
| Systolic blood pressure* | **1.02 (0.99-1.04), 0.084** | 1.02 (0.99-1.04), 0.216 | 1.02 (0.98-1.05), 0.350 | 1.01 (0.99-1.04), 0.299 | 1.03 (0.99-1.06), 0.137 | 1.001 (0.97-1.03), 0.931 |
| Total cholesterol* | 1.07 (0.78-1.47), 0.668 | 1.24 (0.84-1.83), 0.280 | 1.27 (0.78-2.06), 0.342 | 1.28 (0.80-2.07), 0.307 | 1.22 (0.59-2.52), 0.583 | 1.16 (0.49-2.70), 0.740 |
| mRS* | 0.76 (0.47-1.24), 0.270 | 0.77 (0.41-1.45), 0.419 | 0.76 (0.34-1.68), 0.494 | 0.90 (0.51-1.59), 0.709 | 0.57 (0.20-1.62), 0.292 | 1.45 (0.70-3.00), 0.318 |
| Stroke as qualifying event | 0.84 (0.47-1.49), 0.543 | 0.61 (0.29-1.27), 0.186 | 1.14 (0.43-3.01), 0.795 | 0.87 (0.39-1.94), 0.729 | 1.08 (0.32-3.61), 0.901 | 0.75 (0.23-2.43), 0.630 |
| ARWMC | 0.99 (0.89-1.09), 0.986 | 0.94 (0.81-1.09), 0.424 | 1.03 (0.89-1.19), 0.718 | **1.13 (0.99-1.29), 0.069** | **1.21 (0.99-1.48), 0.055** | 1.08 (0.88-1.33), 0.449 |
| Ipsilateral stenosis† | 0.77 (0.22-2.65), 0.673 | 0.41 (0.11-1.53), 0.184 | 0.38 (0.07-1.98), 0.251 | 0.41 (0.09-1.92), 0.260 | 22.75 (0.00-3.9x10^7^), 0.670 | 0.35 (0.04-3.14), 0.349 |
| Contralateral stenosis‡ | 1.22 (0.79-1.87), 0.75 | 0.64 (0.30-1.39), 0.262 | 1.30 (0.67-2.52), 0.441 | 0.64 (0.24-1.73), 0.382 | 0.52 (0.10-2.85), 0.455 | 0.52 (0.09-2.84), 0.453 |

Data are unadjusted Cox regression hazard ratios and 95% confidence intervals in parentheses. Abbreviations: ARWMC = age-related white matter changes; CHD =Coronary heart disease; PAD = Peripheral artery disease; mRS = modified Rankin Scale; *At randomisation. †Ipsilateral degree of stenosis (50-69%, 70-99%). ‡Contralateral degree of stenosis (0-49%, 50-69%, 70-99%, occluded). P values <0.01 are highlighted in boldface.
